# Interleukin-13 gene polymorphisms at −1055 C/T and +2044 G/A positions in patients with squamous cell carcinoma of head and neck

**DOI:** 10.5935/1808-8694.20120010

**Published:** 2015-11-20

**Authors:** Bijan Khademi, Seyed Basir Hashemi, Abbass Ghaderi, Ali Shahrestani, Mohammad Mohammadianpanah

**Affiliations:** a Dr. (Professor of Otolaryngology, Department of Otolaryngology and Head and neck surgery, Khalili Hospital, Shiraz University of Medical Sciences, Shiraz, 71936-13511, Iran); b Dr. (Associate Professor of Otolaryngology, Department of Otolaryngology and Head and neck surgery, Khalili Hospital, Shiraz University of Medical Sciences, Shiraz, 71936-13511, Iran); c Dr. (Professor of Immunology, Cancer research center, Shiraz University of Medical Sciences, Shiraz, Iran); d Dr. (Student Research Committee, Resident of Otolaryngology, Department of Otolaryngology and Head and neck surgery, Khalili Hospital, Shiraz University of Medical Sciences, Shiraz 71936-13511, Iran); e Dr. (Shiraz Institute for Cancer Research, Department of Radiation Oncology, Namazi Hospital, Shiraz, Iran, Postcode: 71936-13511, Iran). Cancer research center, Shiraz University of Medical Sciences, Shiraz, Iran

**Keywords:** carcinoma, genetic, head and neck neoplasms, il-13, polymorphism, squamous cell

## Abstract

Squamous cell carcinoma (SCC) is the most common malignancy that involves the upper aerodigestive tract. Interleukin-13 (IL-13) is an immunoregulatory cytokine that has been reported to have some polymorphisms in it gene associated with same disease especially asthma and allergy. The present study aimed to investigate whether the polymorphisms of IL-13 gene (at positions of 1055C/T in the promoter of 1L-13 gene and +2044G/T exon-4) differ in patients with head and neck SCC from healthy controls.

**Methods:**

This study was investigated in patient with head and neck SCC (n = 137) and age- and sex-matched healthy controls (n = 127). Genotyping of IL-13 gene polymorphism was performed using polymerase chain reaction-based restriction fragment length polymorphism method.

**Results:**

No statistically significant differences were found in the frequencies of genotypes and alleles between patients and control group at both sites (*p* = 0.16). In addition, no association was observed between investigated genotypes and other potential prognostic factors such as age, sex, primary tumor site, tumor size and smoking.

**Conclusion:**

This study suggests that there is no association between IL-13 gene polymorphisms (at position −1055C/T and +2044GI A) and susceptibility of the patients to SCC of head and neck.

## INTRODUCTION

Cancer is a complex genetic disease derived from the accumulation of various genetic changes. Head and neck squamous cell carcinoma (HNSCC) is the most common malignancy of the aerodigestive tract and it accounts for more than 90% of malignancies in this area, with 500,000 new cases word wide^1^. It is the fifth leading cause of cancer incidence and sixth leading cause of cancer-related deaths despite significant improvement in conventional therapies. The overall survival for patients with HNSCC remained poor in past 30 years^2^. HNSCC affects more men than women and the most important independently risk factors are tobacco and alcohol^1^, although susceptibility (immunologic factors and age), environmental and occupational factors may also play a role. Genetic predisposition to SCC is well recognized. Deletion, allelic imbalances or loss of heterozygosity on the short arm of chromosome 3 has been associated with a more aggressive biologic behavior^3^. SCC of the head and neck is associated with production of pro-inflammatory and proangiogenic cytokines. *In vitro* analysis of cell lines derived from HNSCC and *in situ* studies of the local head and neck tumor microenvironment have demonstrated that head and neck tumors express a number of autocrine and paracrine growth factors and cytokines^4^.

The human IL-13 gene is together with the IL-4 gene located within 15 kb on chromosome 5q31^5^. IL-4 and IL-13 share many (but not all) functions. To date, variation screening has been extensively performed for these genes and several single-nucleotide polymorphisms (SNPs) have been found to show functional significance and to be strongly associated with immune-related disease. Recently, six novel polymorphisms have been described for the IL-13 locus including a polymorphism in exon 4 (+2044G/A) leading to an amino acid substitution at position 130 (Arg130Gln), the Gln form is strongly associated with increased serum IgE levels in American and German populations^6^. Another polymorphism is located in promoter region of IL-13 gene (-1055 C/T) which is altered regulation of IL-13 production and increased binding of nuclear proteins^7^. The present study aimed to investigate association of polymorphisms of IL-13 gene (at positions of 1055C/T in the promoter of 1L-13 gene and +2044G/T exon-4) in patients with HNSCC.

## METHOD

A group of 137 patients (mean age of 57.7 years) with head and neck SCC (115 male and 22 female) were recruited in this study and been compared with 127 age- and sex-matched healthy controls (mean age of 57.1 years) whom none had history of cancer and autoimmune disease in their first degree relatives. (Table 1).

**Table 1:** Patients' characteristics.

Mean age (years)	
Cancer patients	57.7 (14-81)
Healthy controls	57.1 (15-80)
Male/Female ratio	
Cancer patients	115/22
Healthy controls	107/20
Primary site	
Larynx	75
Tongue	24
Sinonasal tract	13
Oral cavity	10
Hypopharynx	6
Lip	4
Oropharynx	3
Nasopharynx	2

### DNA preparation

DNA was extracted from peripheral blood samples using the traditional Proteinase-K method. Then DNA was prepared in 300 ng/μl concentration.

### Genotyping assay

Genotyping at both position +2044 and −1055 of IL13 were studied using polymerase chain reaction (PCR)-based restriction fragment length polymorphism method. To evaluate the 1055C/T position, the primer sequences of 5' -ACTTCTGGGAGTCAGAGCCA-3' (forward); and 5'-TACAGCCATGTCGCCTTTTCCTGCTCTTCCGTC-3' (reverse) were used. PCR was carried out in a volume of 15 μl containing 75 ng of genomic DNA, 1.5 mM KCL buffer lX, 1.5 mM MgC12, 0.3 mM dNTPs, 3 pm each primer and 1.2 unit of Taq DNA polymerase. The DNA was first denatured at 94°C for 5 min, then the cycling temperature was set at 94°C for 30 seconds as the first denaturation cycle, 65.5°C for 30 seconds as the annealing cycle, 72°C for 30 seconds as the extension cycle, all repeating for a total 30 cycles, followed by a final extension at 72°C for 10 min. 5 μl of PCR products were electrophoresed on 1.5% agarose gels and visualized with ethidiom bromide staining and Gene documentation. Enzyme digestion was carried out in a 15 μl final volume using 0.5 μl “Hpy199I” enzyme, 1.5 μl NTB4 buffer, 0.15 μl BSA buffer, 2.85 μl H20 and 10 μl of PCR product. The reactions were incubated at 37°C for 2 hours. The sizes of corresponding fragments were: CC = 339, 33 bp, CT = 372, 339, 33 bp and TT = 372 bp. Finally, the digested PCR products were electrophoresed on 2.7% agarose gels to separate the fragments The assay conditions for the polymorphism at +2044G/A was as following: forward primer: 5'-CTTCCGTGAGGACTGAATGAGACGGTC-3' and reverse primer: 5'-GCAAATAATGATGCTTTCGAAGTTTCAGTGGA-3', each was used for a total of 6 pm per a tube. The “NlaIV” restriction enzyme was used to digest 236 bp fragment and produce GG = 178, 32, 26 bp, GA = 216, 178, 32, 26 bp and AA = 216, 26 bp fragments. The optimal annealing temperature for this test was 62.5°C.

### Statistical analysis

Allele and genotype frequencies and quantitative traits were compared between control and patients' subtypes by the use of Chi-square (χ^2^) and Fisher exact tests. The results were then scheduled by SPSS 15 and EPI info (version 2002) soft wares and the associations were analyzed to adjust for different respective factors. P-values more than 0.05 were considered insignificant.

This study was approved by the Ethics Committee of the institution.

## RESULTS

### IL-13, −1055 and +2044 genotypes and alleles

As depicted in Table 2, screening of the patients for the IL-13 genotype and allele frequencies of the C-l055T and G+2044A polymorphisms revealed no significant differences in these two genotypes and their alleles in patients with HNSCC compared to healthy controls. Different genotypes of both sites were also collated two by two to evaluate any combination through them. According to the data depicted in Table 3, no linkage was identified within either of 137 patients and 127 control subjects.

**Table 2:** Allele and Genotype frequencies at −1055C/T and +2044 G/A positions in IL-13.

Subjects	-1055C/T					+2044G/A				
Genotype	CC	CT	TT	C allele	T allele	GG	GA	AA	G allele	A allele
Patient (n = 137)	86	41	7	211	59	73	56	8	200	72
	(62%)	(30%)	(8%)	(78%)	(22%)	(53%)	(41%)	(6%)	(74%	(26%)
Control (n = 127)	61	48	8	171	67	53	51	13	157	79
	(48%)	(37%)	(15%)	(72%)	(17%)	(42%)	(41%)	(17%)	(67%)	(33%)
*p*-value	0.16			0.1		0.17			0.08

**Table 3:** Genotype distribution at −1055C/T and +2044 G/A positions in IL-13 among patients with HNSCC and healthy controls.

Subjects	CC+GG	CC=GA	CC+AA	CT+GG	CT+GA	CT+AA	TT+GG	TT+GA	TT+AA
Patient (n = 137)	52	30	3	17	24	3	3	2	2
	(38%)	(22%)	(2%)	(13%)	(18%)	(2%)	(2%)	(1.5%)	(1.5%)
Control (n = 127)	34	20	4	15	28	6	3	3	3
	(27%)	(16%)	(3%)	(12%)	(22%)	(5%)	(2%)	(2%)	(2%)
*p*-value					0.62				

### IL-13 −1055 and +2044 genotypes and head and neck predisposing factors

Genotypes and alleles harvested from the two polymorphisms were investigated in order to achieve any association with patients' baseline values like age and gender or prognostic/predictive factors such as primary tumor site, tumor size and smoking. Although, HNSCC is consistently attributed to each of them, no association was observed regarding these two polymorphism substitutions.

## DISCUSSION

Cancer genes are generally grouped into oncogenes and tumor suppressor genes. Neoplastic diseases are complex genetic disorder that results from imbalance between activation of proto-oncogenes and inactivation of tumor suppressor genes. The human genome project currently offers a unique opportunity for identification of all the genetic changes associated with a human cancer^8^. Cytokines and related genes can directly or indirectly act against defense mechanisms of tumor with changes in type of immune response and its quality ^2^. T helper immune responses or cytotoxic T cell activation are essential for antitumor actions. Several studies with animal model and human patients with cancer revealed that in these cases conventional responses of Th1 and activities of CD8+ cells were inhibited. The tendency of antitumor immune responses to Th2 leads to suppression of Th1 responses^9^^,^^10^. In a study, the expression of Th1 and Th2 cytokines was evaluated in human tumoral cells that revealed dominancy of type II cytokines in 20 types of tumor cells. The escape of tumor cells from immune control may be related to this phenomenon^10^.

In other studies, the investigators observed that IL-4 and IL-13 can inhibit the tumor regression^11^. In another study, The effects of human IL-4 and IL-13 on clonal growth of breast cancer cells were investigated that revealed IL-13 can reduced clonal growth of 3 cell type^7^. These studies concluded that IL-13 have important role in regulation of antitumor activities. There were several polymorphisms in IL-13 gene. The 1055C/T was located in promoter region of IL-13 gene. The change of C to T nucleotide in this point may alter attachment and transcription factor type and then alter the expression of IL-13 gene. Several studies revealed that −1055C/T polymorphism was associated with allergic asthma, altered regulation of IL-13 production and increased binding of nuclear protein^12^.

Another important polymorphism in IL-13 gene is +2044 G/A that is located on Exon-4 that is important region for receptor-ligand interaction. Replacement of Arginin to Glutamine (Arg130G1n) leads to creation of an IL-13 protein with high affinity to its receptor. This can explain theoretically many aspects of allergic phenotype (IgE production, mucus secretion, eosinophilic inflammation and hyper sensitivity reaction)^9^. This allele was associated with increased susceptibility of diabetes type 1 and COPD and its frequency was low in patients with Juvenile idiopathic arthritis and graves disease^3^. In our study the 1055C/T and +2044G/A polymorphisms was analyzed in patients with head and neck SCC.

Previous study has been carried out on patients with head and neck SCC.

In the literature, there are few studies investigating the association of IL-13 haplotypes and susceptibility to cancers. Faghih et al.^13^ investigated the association of IL-13 haplotypes and susceptibility of Iranian women to breast cancer. They found no significant differences in the distribution of −1512A/C, −1055C/T and +2044G/A genotypes or alleles between patients and the control group; however, they suggested a potential association of CCA haplotype of IL13 gene with susceptibility of Iranian women to breast cancer. In another study, Sameni et al.^14^ found no association between interleukin-13 gene polymorphisms (-1055 C/T and +2044 G/A) in Iranian patients with lung cancer. To the best of our knowledge, this is the first study investigating IL-13 gene polymorphisms at −1055 C/T and +2044 G/A positions in patients with squamous cell carcinoma of head and neck.

## CONCLUSION

Results of this study indicate no association between IL-13 gene polymorphisms (at position −1055C/T and +2044G/A) and susceptibility of the patients to HNSCC.
